# Effects of vibroacoustic stimulation in music therapy for palliative care patients: a feasibility study

**DOI:** 10.1186/s12906-015-0933-8

**Published:** 2015-12-15

**Authors:** Marco Warth, Jens Kessler, Svenja Kotz, Thomas K. Hillecke, Hubert J. Bardenheuer

**Affiliations:** Centre of Pain Therapy and Palliative Care Medicine, Department of Anaesthesiology, University Hospital Heidelberg, Im Neuenheimer Feld 131, 69120 Heidelberg, Germany; School of Therapeutic Sciences, SRH University Heidelberg, Maria-Probst-Strasse 3, 69123 Heidelberg, Germany

**Keywords:** Music therapy, Palliative care, Feasibility, Singing chair, End-of-life care, Advanced cancer patients, Heart rate variability

## Abstract

**Background:**

The present study aimed at examining whether methodological strategies from a previously implemented study design could be transferred to the evaluation of the psychological and physiological effects of a music therapy intervention working with vibroacoustic stimulation in palliative care.

**Method:**

Nine participants suffering from advanced cancer took part in single-sessions of music therapy, lasting for 30 min. The live music therapy intervention utilized singing chair sounds and vocal improvisation. Visual analogue scales (VAS) were used to assess self-ratings of pain, relaxation, and well-being before and after each session. During the intervention, we continuously recorded heart rate variability (HRV) as a measure of autonomic functioning. Data collection was complemented by a semi-structured interview to explore subjective experiences in more detail. Feasibility was defined as the ability to complete 80 % of the sessions in accordance with the study protocol.

**Results:**

In 5 out of 9 sessions (55 %) it was possible to deliver the intervention and obtain all data as intended. VAS assessment was feasible, although graphical and statistical examination revealed only marginal mean changes between pre and post. HRV recordings were subject to artifacts. While HRV parameters differed between individuals, mean changes over time remained relatively constant. Interview data confirmed that the individual perception was very heterogeneous, ranging from “calming” to “overwhelming”.

**Conclusion:**

The criterion of feasibility was not met in this study. Physiological data showed high attrition rates, most likely due to movement artifacts and reduced peripheral blood flow in some participants’ extremities. Examination of individual-level trajectories revealed that vibroacoustic stimulation may have an impact on the autonomic response. However, the direction and mechanisms of effects needs to be further explored in future studies.

**Trial registration:**

German Clinical Trials Register – DRKS00006137 (July 4^th^, 2014).

## Background

Music therapy is defined as the “[…] systematic process of intervention wherein the therapist helps the client to promote health, using music experiences and the relationships that develop through them as dynamic forces of change” [1] and thereby contrasts ‘music medicine’ or mere ‘music listening’ interventions that do not require the presence of a professional therapist. During the last decades, music interventions have increasingly been implemented as complementary treatments in different medical settings, such as oncology, intensive or palliative care [2–4]. The World Health Organisation defines palliative care as “[…] an approach that improves the quality of life of patients and their families facing the problems associated with life-threatening illness” [5]. Palliative care holds a holistic and multidisciplinary perspective on the patients’ and family caregivers’ needs. Hence, alternative and complementary treatments such as music therapy have been integrated into palliative care since the foundation of the first institutions in the late 1970’s [6].

Today, the work of music therapists receives high acceptance by other health-care professionals in end-of-life care [7], and music therapy interventions are among the most frequently used complementary treatments in US hospices [8, 9]. The aim of music therapy in this field is to improve or maintain the patient’s quality of life by supporting physical symptom management (e.g. pain, stress symptoms, shortness of breath), addressing emotional needs, enhancing communication, and facilitating spiritual experiences [10]. Families and caregivers may participate in the therapeutic process, and music therapy programs can address the needs of bereaved family members during feelings of loss and grief [11, 12]. The most common interventions encompass the use of improvisation and songs, as well as receptive relaxation and imagery techniques [10]. In contrast to active music therapy - where the patient himself plays an instrument or uses his voice – receptive techniques require very little or no physical activity and mental strain, and thus, are frequently used in end-of-life care settings.

Among relaxation and imagery techniques, the application of monotone instruments and vibroacoustic stimulation has increasingly gained importance during the past years [13]. One possible rationale for the use of vibroacoustic stimulation in medical settings is the idea that musical and emotional experiences may be intensified if a person not only listens to music, but actually senses the tactile vibration. Therapeutic instruments making use of this phenomenon are the monochord [14], body tambura [13], and the singing chair [15].

Despite its long tradition and high acceptance in palliative care, empirical research on the effects of music therapy is still rare. A recent review concluded that the available evidence is not sufficient to give a valid recommendation in favor or against the use of music therapy in this field [16]. The 2010 Cochrane review (withdrawn from the database in 2014) included five controlled clinical trials that showed small to moderate effects of music therapy interventions on quality of life, pain, as well as emotional and spiritual well-being [4, 17–21]. Further pilot studies showed first evidence that music therapy may be effective in facilitating communication [22] and reducing cortisol stress level [23]. However, the risk of bias in most studies was high, due to small sample sizes, a lack of control conditions, unclear randomization and concealment methods, or insufficient statistical reporting [4, 24]. A recently conducted randomized controlled trial (RCT) implemented a more rigorous research design and showed that music therapy reduced subjective ratings of pain intensity among 200 hospitalized patients with advanced cancer. Another RCT is currently being accomplished by our own research group [25].

Feasibility studies form an important step towards the implementation of larger-scale experimental research, as they offer the opportunity to evaluate which outcome measures are most suitable to quantify the effect, and to identify inadequate methodological ideas. Particularly, in challenging and sensitive research settings such as end-of-life care, they may help to prevent researchers from implementing clinical trials that might not be feasible or capable of producing valid results [26].

One goal of the previous RCT [25] was to explore possibilities for follow-up studies. Therefore, the aim of the present pilot study was to investigate, whether it was feasible to transfer elements of the previous research design to the evaluation of the effects of vibroacoustic stimulation for advanced cancer patients in palliative care.

## Methods

### Participants and procedures

The study received ethical approval by the Ethics Committee of the Medical Faculty at Heidelberg University (S - 406/2012). All participants were contacted and recruited at the palliative care unit of St. Vincencius Hospital in Heidelberg, Germany. The inclusion and exclusion criteria listed in Table 1 were assessed to determine eligibility for study participation.

**Table 1:** Inclusion and exclusion criteria

Inclusion criteria	Exclusion criteria
Receiving palliative care	Terminal phase
Sufficient understanding of German or English language	Cognitive impairments (e.g. brain metastases, ICD 10: C71, C72)
	Apallic syndrome (ICD 10: G93.80)
	Deafness (ICD 10: H90, H91)
	Restlessness and agitation (ICD 10: R45.1)
	Immobility (unable to sit in an upright position for 30 min)

The present study utilized a single-group pre-post mixed-methods design with continuous recordings of physiological data. Participants eligible for study inclusion were contacted in their rooms, were informed about the study objectives, and asked to sign the informed consent sheet. If a patient expressed willingness to participate, the investigator left the room and shortly after returned together with the music therapist, the singing chair, and the measuring devices. After baseline assessment of relaxation, well-being, and pain via visual analogue scales (VAS) and a five-minute baseline recording of heart rate variability (HRV), the investigator left the room and the music therapist initiated the intervention. Twenty minutes later, the investigator returned for post-test assessment of VAS and HRV, followed by a semi-structured qualitative interview on subjective experiences during the intervention. The entire session duration was approximately 30–35 min.

### Intervention

A singing chair was used for live musical play in the music therapy relaxation intervention. A singing chair is a musical instrument designed for therapeutic purposes by instrument maker Bernhard Deutz^1^. At the backside of the instrument, 69 steel strings are vertically placed in tuning of the traditional Indian tambura (i.e. strings were tuned sequentially in the notes A-d-d-D). A recent pilot study found that after five minutes of a singing chair intervention, apparently healthy students showed a reduction in heart rate and anxiety [15]. In a qualitative interview study, terminally-ill patients reported to feel more relaxed and calm, and to have experienced pleasant body sensations and visualizations after a music therapy session using the body tambura (an instrument with similar characteristics) [13].

The intervention was carried out by a music therapist with several years of experience in working with terminally-ill patients. The musical intervention started with a short body scan (i.e. a mindfulness exercise focusing attention on different body parts) accompanied by slow and gentle play of the backside strings, lasting for approximately 3 min. The therapist then initiated a vocal improvisation in an ionian or mixolydian mode (i.e. diatonic musical keys defined by a sequence of whole steps and half steps), while she gradually increased the volume, dynamics, and range of her musical play. During this improvisation, the music therapist tried to synchronize and modify musical and breathing meter. After 10–12 min, the intensity of music and singing were slowly reduced and finally faded out. The therapist gently asked the participant to return his attention to the present moment. Afterwards the patient had the opportunity to reflect on his subjective experiences during the singing chair music.

### Outcomes

Three VAS ranging from 0 to 10 were used to assess pre and post measures of patients’ subjective experience of pain, relaxation, and well-being. The scales’ endpoints were illustrated with good vs. bad smileys and contained a colour code from red to green.

As a peripheral measure of autonomic function, we continuously recorded the intervals between successive heart beats in milliseconds (i.e. HRV). High frequent (HF) oscillation in these inter-beat-intervals (IBI) is indicative of parasympathetic activity, and is controlled predominantly via the vagus nerve [27]. Thus, a relaxation response [28] was expected to manifest in a decrease in absolute heart rate (mean HR) and in an increase of high frequent variation in heart rate over time (HF power). We used a NeXus-16 Bluetooth interface together with a photoplethysmography finger sensor for non-invasive measurement of HRV. The signal was recorded by BioTrace+ software on a laptop computer inside the patient’s room. IBI tables for the entire session duration were exported for each participant. Kubios v2.1 was used to extract six successive time segments of 5 min duration for further analyses with IBM SPSS Statistics 20 for each session.

Since no evidence exists on the effects (and possible side effects) of the use of singing chair interventions in end-of-life care, we added a semi-structured interview with an open answer format to explore the assumed mechanisms of the intervention in more detail. The questions focused on spontaneous reactions, physical sensations, cognitive perceptions, and subjective feelings after the intervention, as well as on the comparison of the perceived effects to similar relaxation exercises.

### Analysis

Answers to open-ended interview questions were interpreted according to content analysis [29]. Statistical analysis of HRV and VAS data was performed by using IBM SPSS Statistics 20. Since we expected a small sample size in this pilot study, the analysis focused on descriptive statistics (means and standard deviations) and confidence intervals (CI). For reason of completeness, *p*-values from repeated-measures analysis of variance (RM-ANOVA) were calculated. Feasibility was defined as the ability to complete 80 % of the single sessions in accordance with the study protocol. We attempted to recruit 10 participants for this study.

## Results

**Fig. 1:**
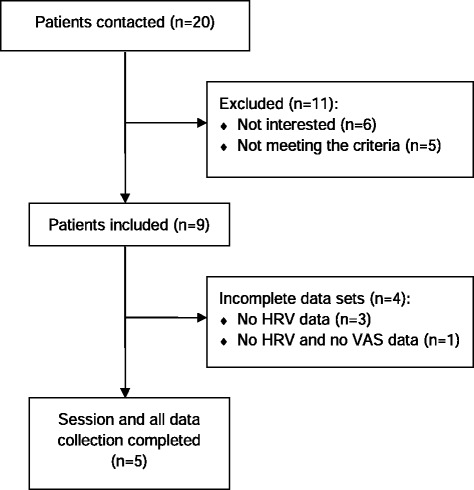
Patient flow chart

Within the available amount of time (March to July 2014), it was possible to include a total of 9 participants (6 female) with a mean age of 59.9 (±7.15) years in this study. All participants suffered from progressive, life-threatening cancer: liver carcinoma (*n* = 3), metastatic melanoma (*n* = 1), prostatic carcinoma (*n* = 1), leukemia (*n* = 1), angiosarcoma (*n* = 1), gastric cancer (*n* = 1), and pancreatic cancer (*n* = 1). The average Karnofsky index (i.e. a rating system on functional impairment) was rated *M* = 45.0 %, *SD* = ±10.7 % (mean ± standard deviation), indicating that the participants on average were in need of special care and assistance. The most frequently reported symptoms among all participants were immobility, pain, fatigue and loss of appetite, as rated by the physician in charge. Figure 1 depicts a flow diagram and shows that in 5 out of 9 participants (56 %) the intervention and study protocol could be delivered as intended.

**Fig. 2:**
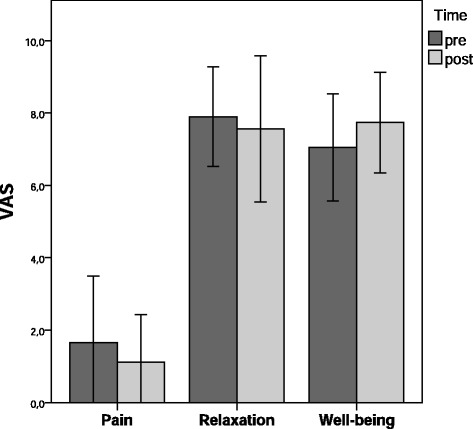
Pre to post comparison of VAS (*N* = 8). Means and 95 % confidence intervals

VAS data collection was completed in 8 of 9 participants. One participant showed signs of severe exhaustion immediately after the intervention and was not able to respond to post-test questions. Figure 2 shows mean values and 95 % confidence intervals (CI) for pre and post VAS scores. For all three variables, patients showed very little change over time. Marginal improvements were observed for pain (mean difference, *MD* = -0.54) and well-being (*MD* = 0.69), and a minimal decline occurred for relaxation (*MD* = -0.33). However, CIs were highly overlapping and RM-ANOVA did not detect any statistically significant differences (all *p* > .05; see Table 2).

**Table 2:** Changes in mean (±SD) VAS scores from pre- to post-intervention (*N* = 8)

	Pre	Post	MD	CI of MD	p*
Pain	1.65 (2.21)	1.11 (1.58)	−0.54 (1.17)	[-0.44, 1.51]	.23
Relaxation	7.90 (1.64)	7.56 (2.41)	−0.33 (2.05)	[-1.37, 2.05]	.66
Well-being	7.05 (1.77)	7.74 (1.66)	0.69 (2.41)	[-2.70; 1.32]	.45

MD = mean difference, CI = 95 % confidence interval

*p-values from RM-ANOVA (considered significant, if *p* < .05)

**Fig. 3:**
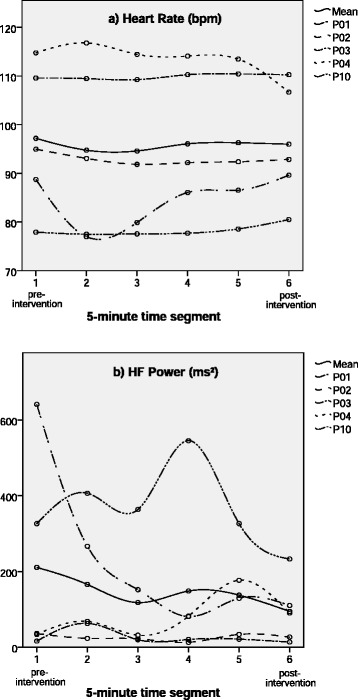
Individual and mean changes of HRV parameters over time (*N* = 5). **a** Heart Rate, (**b**) HF Power

For only 5 of the 9 sessions, a complete 30-min HRV data set was recorded and could be used for further analysis. Two sessions had to be excluded due to movement artifacts, and in another two cases the recordings were biased, most likely because of severely reduced peripheral blood flow. Because of the very small database, Figs. 3a and 3b contain patients’ individual trajectories in addition to the mean variation over time (solid line). Graphical examination did not reveal relevant changes in mean HR. Only one participant (P01) showed the expected decrease in HR when the actual intervention started (time segment 2). Regarding vagally-mediated HRV (HF power), baseline values and time courses differed extensively between participants. While P10 showed the expected quadratic trajectory (increase during the intervention, decrease to baseline afterwards), the HF component of P1 strongly declined. All other participants’ values did not change considerably over time. As Table 3 indicates, none of the tested quadratic trends (as well as linear and cubic trends) was statistically significant (all *p* > .05).

**Table 3:** Changes in mean (±SD) HR and HF over time (*N* = 5)

	Pre	Intervention				Post	
	T1	T2	T3	T4	T5	T6	p*
HR	97.19	94.75	94.60	96.06	96.28	95.98	.50
	(15.09)	(18.16)	(16.76)	(15.64)	(15.17)	(12.34)	
HF	210.74	165.59	117.77	148.27	137.53	94.60	.81
	(273.19)	(164.91)	(148.24)	(224.55)	(124.16)	(87.43)	

T = 5-min time segment, HR = heart rate [bpm], HF = power in the high frequency spectrum [ms^2^]; *p-values for quadratic trends over time in RM-ANOVA (considered significant, if *p* < .05)

It was possible to complete six semi-structured interviews, as three participants experienced symptoms of fatigue or tiredness after the intervention, and were not able to answer all of the questions. Spontaneous reactions of respondents to the experienced singing chair intervention were heterogeneous and ranged from “slightly sceptic” to “overwhelming” and “enthusiastic”. Two of the research subjects perceived physical changes and reported improvement in breathing and relaxation of the muscles. Regarding cognitive changes, three patients reported that they felt sleepier than before the onset of the intervention. One respondent reported an emergence of negative visualisation without feeling of a strong emotional arousal. Four respondents felt calmer and more relaxed, although one patient reported to be more aroused afterwards. Compared to previous experience with relaxation methods, one participant rated the singing chair intervention as less effective, two participants were not able to find any qualitative differences and three participants had not attended any relaxation exercises before.

## Discussion

The primary aim of this feasibility study was to examine whether the procedures and outcomes from a recently implemented study [25] could be transferred to the evaluation of a newly developed singing chair intervention in palliative care. Feasibility was defined as the ability to complete 80 % of the interventional sessions in accordance with the study protocol. This criterion was not met, as only 5 out of 9 participants (56 %) completed all data including psychological and physiological measures. Recruitment of participants was difficult and time-consuming. Many patients were not eligible for study participation due to the exclusion criteria (Table 1). Particularly, the requirement to sit in an upright position for up to half an hour led to a reduction in the number of eligible subjects.

VAS were used to measure participants’ subjective feeling of pain, well-being and relaxation before and after the intervention. Graphical and statistical analysis showed no relevant mean differences between pre- and post-values in any of the three outcomes. The largest improvement occurred for well-being with a mean difference of *MD* = 0.69 (±2.41). This contradicts findings from our previous study using a monochord intervention at bedside, where we found mean changes from pre to post of up to two points on a VAS with a larger sample size of *N* = 84 [30]. However, it would be false to conclude that the singing chair intervention does not affect these three dimensions, in general. Examination of the individual values revealed rather favorable baseline scores for pain (*M* = 1.65, *SD* = 2.21), relaxation (*M* = 7.90, *SD* = 1.64), and well-being (*M* = 7.05, *SD* = 1.77), giving a possible explanation for the lack of observable improvements. While the majority of terminally-ill patients in palliative care suffer considerably from symptoms such as pain, stress, and anxiety [31], the relatively strict inclusion criteria in the present study obviously led to a highly selective, non-representative sample. Regarding feasibility, however, the VAS proved to be manageable and economic measures of current subjective states. For VAS on acute pain, previous studies found evidence for adequate reliability and validity [32, 33].

Only minor changes were observed in the graphical analysis of mean HRV parameters. However, important inferences can be drawn from the individual trajectories displayed in Figs. 3a and 3b: The variability between individual intercepts and slopes seemed to have a higher impact than the mean changes over time, resulting in the mean curve not adequately representing the majority of individual trajectories. This issue appears to apply to many statistical analyses on physiological data as a study outcome, and has been addressed recently in a methodological paper, suggesting the integration of more ideographic approaches to data analysis in such cases [34]. Growth curve models (or multilevel analyses) seem to be suitable as they allow for characterizing both group-level and individual-level effects, accounting for inter-individual differences in regression intercepts and slopes.

The relatively high percentage of HRV measuring artifacts in the present study of 44 % is not representative of our research experience in palliative care in general, where we had attrition rates between 10 % and 20 % for 30-min of HRV recording. However, the need to sit in an upright position might have contributed to movement artifacts and erroneous recordings. We would, however, still reject the alternative to use multi-point electrocardiogram (ECG) recordings, as electrode placement may require too much strain for some patients in palliative care.

Analysis of the semi-structured interviews is accordant with findings from the examination of individual HRV scores, illustrating that – in contrast to a monochord intervention – the direction of effects in a singing chair intervention cannot yet be predicted in a general manner. Although most patients perceived the sounds and tactile stimulation as positive and calming, some patients reported experiences of arousal or being overwhelmed.

The present investigation was designed as a pilot feasibility study, and hence, faced several methodological limitations. Firstly, we did not implement randomization or a control condition. The reason was that our previous study successfully utilized a pre-recorded mindfulness exercise as an active and ethically sound control intervention. If a larger-scale RCT was to be designed on the basis of findings from the present pilot study, this mindfulness exercise may be used as a control condition. Secondly, we included a small and non-representative sample, impeding the possibility to draw causal inferences on the effectiveness of vibroacoustic stimulation from the presented statistical tests. Finally and as discussed above, the inclusion and exclusion criteria used in this pilot study showed to be problematic in ways that they significantly limited the number of eligible patients and that they were in part imprecisely defined. We would therefore recommend the use objective indicators for criteria such as “cognitive impairment” or “final phase” in future studies (e.g. use of the Karnofsky scale).

## Conclusion

Music therapy is a frequently used complementary therapeutic approach in end-of-life care. In response to the attested lack of high-quality research in this field [4, 16], attempts have been initiated to create an evidence-based rationale in recent years [25, 35]. The present study explored whether methodological strategies from a previously implemented research design could be transferred to the evaluation of a singing chair intervention for hospitalized patients in a palliative care unit. Results suggest that the chosen design integrating both psychological and physiological outcomes in the context of a strictly standardized protocol was not feasible for the population and intervention under investigation. This, however, does not imply that interventions using vibroacoustic stimulation are not effective in improving patients’ quality of life or subjective well-being. Instead we recommend exploring adequate endpoints and possible working mechanisms in future qualitative and mixed-methods studies prior to the implementation of larger-scale effectiveness research.

## Abbreviations

CI: Confidence interval
ECG: Electrocardiogram
EORTC: European Organization for Research and Treatment of Cancer
HF: High frequency power spectrum
HR: Heart rate
HRV: Heart rate variability
IBI: Inter-beat-interval
M: Mean
MD: Mean difference
RCT: Randomized controlled trial
RM-ANOVA: Repeated-measures analysis of variance
SD: Standard deviation
SMD: Standardized mean difference
VAS: Visual analogue scale

## Footnotes

1 http://www.deutz-klangwerkstatt.de/instrumente/klangstuhl

## References

[1] Bruscia KE (1998). Defining Music Therapy.

[2] Bradt J, Dileo C (2014). Music interventions for mechanically ventilated patients. Cochrane Database Syst Rev.

[3] Bradt J, Dileo C, Grocke D, Magill L (2011). Music interventions for improving psychological and physical outcomes in cancer patients. Cochrane Database Syst Rev.

[4] Bradt J, Dileo C (2014). WITHDRAWN: Music therapy for end-of-life care. Cochrane Database Syst Rev.

[5] Watson M, Lucas C, Hoy A, Wells J (2009). Oxford handbook of palliative care.

[6] Munro S, Mount B (1978). Music therapy in palliative care. Can Med Assoc J.

[7] O'Kelly J, Koffman J (2007). Multidisciplinary perspectives of music therapy in adult palliative care. Palliat Med.

[8] Bercovitz A, Sengupta M, Jones A, Harris-Kojetin LD (2007). Complementary and Alternative Therapies in Hospice: The National Home and Hospice Care Survey: United States. Natl Health Stat Report.

[9] Kozak LE, Kayes L, McCarty R, Walkinshaw C, Congdon S, Kleinberger J, Hartman V, Standish LJ (2008). Use of complementary and alternative medicine (CAM) by Washington State hospices. Am J Hosp Palliat Care.

[10] Warth M, Koenig J, Keßler J, Wormit AF, Hillecke TK, Bardenheuer HJ (2014). Musiktherapie in der palliativmedizinischen Versorgung: Gegenwärtiger Stand und aktuelle Entwicklungen. Musikther Umsch.

[11] Magill L (2009). The meaning of the music: the role of music in palliative care music therapy as perceived by bereaved caregivers of advanced cancer patients. Am J Hosp Palliat Care.

[12] O'Callaghan C, McDermott F, Hudson P, Zalcberg JR (2013). Sound continuing bonds with the deceased: the relevance of music, including preloss music therapy, for eight bereaved caregivers. Death Stud.

[13] Teut M, Dietrich C, Deutz B, Mittring N, Witt CM (2014). Perceived outcomes of music therapy with Body Tambura in end of life care - a qualitative pilot study. BMC Palliat Care.

[14] Lee EJ, Bhattacharya J, Sohn C, Verres R (2012). Monochord sounds and progressive muscle relaxation reduce anxiety and improve relaxation during chemotherapy: a pilot EEG study. Complement Ther Med.

[15] Tanner R: The Effects of Vibroacoustic Music (VAM) in Reducing Performance Level Anxiety in College Students. Utah State University 2012 [http://www.feeltoneusa.com/user-feedback-/ewExternalFiles/Sound%20Chair%20Utah%20Research%20Study.pdf].

[16] Korczak D, Wastian M, Schneider M (2013). Music therapy in palliative setting. GMS Health Technol Assess.

[17] Hilliard RE (2003). The effects of music therapy on the quality and length of life of people diagnosed with terminal cancer. J Music Ther.

[18] Horne-Thompson A, Grocke D (2008). The effect of music therapy on anxiety in patients who are terminally ill. J Palliat Med.

[19] Nguyen J (2003). The effect of music therapy on end-of-life patients’ quality of life, emotional state, and family satisfaction as measured by self-report. Master's Thesis.

[20] Wlodarczyk N (2007). The effect of music therapy on the spirituality of persons in an in-patient hospice unit as measured by self-report. J Music Ther.

[21] Lee H (2005). The Effect of live music via the iso-priniciple on pain management in palliative care as measured by self-report using a graphic rating scale (GRS) and pulse Rate. Master's Thesis.

[22] Brown J (2006). Comparison of the effects of music and conversation on hospice patient’s predisposition to communicate and communication behaviors. Master's Thesis.

[23] Nakayama H, Kikuta F, Takeda H (2009). A pilot study on effectiveness of music therapy in hospice in Japan. J Music Ther.

[24] Warth M, Kessler J, Koenig J, Wormit AF, Hillecke TK, Bardenheuer HJ (2015). Methodological challenges for music therapy controlled clinical trials in palliative care. Nord J Music Ther.

[25] Warth M, Kessler J, Koenig J, Wormit AF, Hillecke TK, Bardenheuer HJ (2014). Music therapy to promote psychological and physiological relaxation in palliative care patients: protocol of a randomized controlled trial. BMC Palliat Care.

[26] Hagen NA, Biondo PD, Brasher PM, Stiles CR (2011). Formal feasibility studies in palliative care: why they are important and how to conduct them. J Pain Symptom Manage.

[27] Task Force of the European Society of Cardiology and the North American Society of Pacing and Electrophysiology (1996). Heart rate variability. Standards of measurement, physiological interpretation, and clinical use. Eur Heart J.

[28] Dusek JA, Benson H (2009). Mind-body medicine: a model of the comparative clinical impact of the acute stress and relaxation responses. Minn Med.

[29] Mayring P (2010). Qualitative Inhaltsanalyse: Grundlagen und Techniken.

[30] Warth M, Kessler J, Hillecke TK, Bardenheuer HJ: Rezeptive Musiktherapie in der Palliativmedizin: Eine randomisiert kontrollierte Studie zur Beurteilung entspannungsfördernder Effekte. *Deutsches Ärzteblatt* in press

[31] Potter J, Hami F, Bryan T, Quigley C (2003). Symptoms in 400 patients referred to palliative care services: prevalence and patterns. Palliat Med.

[32] Gallagher EJ, Bijur PE, Latimer C, Silver W (2002). Reliability and validity of a visual analog scale for acute abdominal pain in the ED. Am J Emerg Med.

[33] Bijur PE, Silver W, Gallagher EJ (2001). Reliability of the visual analog scale for measurement of acute pain. Acad Emerg Med.

[34] Kristjansson SD, Kircher JC, Webb AK (2007). Multilevel models for repeated measures research designs in psychophysiology: an introduction to growth curve modeling. Psychophysiology.

[35] Gutgsell KJ, Schluchter M, Margevicius S, DeGolia PA, McLaughlin B, Harris M, Mecklenburg J, Wiencek C (2013). Music therapy reduces pain in palliative care patients: a randomized controlled trial. J Pain Symptom Manage.

